# Serine protease-driven entry and S2 ′ cleavage flexibility of feline coronavirus during feline enterocyte infections

**DOI:** 10.1371/journal.ppat.1013854

**Published:** 2026-01-08

**Authors:** Bixia Chen, Luna Vanden Buijs, Nathalie Vanderheijden, Lowiese Desmarets, Jolien Van Cleemput, Hans J. Nauwynck

**Affiliations:** 1 Department of Translational Physiology, Infectiology and Public Health, Faculty of Veterinary Medicine, Ghent University, Merelbeke, Belgium; 2 European Partnership on Animal Health and Welfare, Faculty of Veterinary Medicine, Ghent University, Merelbeke, Belgium; 3 CNRS, Inserm, CHU Lille, Institut Pasteur de Lille, U1019-UMR 9017-CIIL-Center for Infection and Immunity of Lille, Université de Lille, Lille, France; Texas Biomedical Research Institute, UNITED STATES OF AMERICA

## Abstract

Coronaviruses not only hijack host cells to serve as viral factories but also exploit host proteolytic systems to activate their spike (S) protein, the key glycoprotein mediating receptor binding and membrane fusion. Feline coronavirus (FCoV), which initially replicates in the intestinal tract, has evolved to utilize local intestinal proteases for S protein activation. This activation occurs through proteolytic cleavage at specific regions on the S protein, known as cleavage sites (CSs). Two putative CSs have been proposed for FCoV: S1/S2 CS and S2′ CS. Through a protease screen, we identified serine proteases as particularly critical for FCoV infection. Notably, three pancreatic serine proteases, chymotrypsin, trypsin, and elastase, enhanced FCoV infection and promoted syncytia formation despite their differing cleavage specificities, suggesting a flexible activation strategy. Furthermore, the membrane-bound serine proteases TMPRSS2 and TMPRSS11D also facilitated infection and syncytia formation in a strain-dependent manner. By analyzing the cleavage profiles of these serine proteases, we experimentally confirmed these two putative CSs on the FCoV S protein and identified additional CSs. Importantly, our analysis revealed a compensatory cleavage mechanism at the S2′ CS that maintains spike activation even when mutations disrupt the canonical cleavage motif, underscoring the central role of S2′ CS in viral infection. Additionally, an acidic microenvironment is required for efficient infection. Together, these findings illustrate how FCoV adapts to locally available serine proteases to optimize S protein priming and intestinal cell entry.

## Author summary

Like many viruses, the common feline coronavirus (FCoV) relies on the host replication machinery to infect intestinal cells. Our study revealed how it works: FCoV exploits intestinal enzymes called serine proteases, which act like molecular scissors to cleave and activate the virus’s spike protein, the “key” the virus uses to unlock and enter cells. Without this activation step, infection cannot occur. The spike protein is cleaved by serine proteases primarily at two sites, S1/S2 and S2′, with S2′ playing a particularly critical role; even if mutations disable S2′, the virus cleverly uses backup sites nearby to remain infectious. Both free-floating serine proteases in the gut and membrane-bound ones on host cells can activate the spike, and blocking these enzymes significantly reduces viral infection. These findings reveal how FCoV takes advantage of its intestinal environment to ensure successful entry, and point to a promising antiviral strategy: targeting the host enzymes the virus depends on.

## Introduction

The intestinal system is a complex and highly dynamic environment that functions not only as a digestive organ but also as a critical immunological barrier. This system harbors a diverse repertoire of proteases essential for maintaining gut health [1]. Based on their catalytic mechanisms, intestinal proteases are classified into cysteine proteases, metalloproteinases, aspartic proteases, and serine proteases [2]. Serine proteases represent a major component of the intestinal proteolytic machinery [3]. Key examples include the pancreatic enzymes chymotrypsin, trypsin, and elastase, which are synthesized as inactive zymogens in the exocrine pancreas and activated in the small intestine. Trypsin is activated by enteropeptidase and the others are activated by trypsin [4,5]. Chymotrypsin cleaves after aromatic amino acids (phenylalanine, tyrosine, and tryptophan), trypsin cleaves after basic amino acids (arginine and lysine), and pancreatic elastase cleaves after small hydrophobic amino acids, such as glycine, alanine, and valine [6].

As a key interface with the external environment, the intestinal tract is continuously exposed to potential pathogens. One such pathogen is feline coronavirus (FCoV), a common virus in cat populations that is typically considered to occur in two pathotypes: feline enteric coronavirus (FECV) and feline infectious peritonitis virus (FIPV). FECV, the intestinal variant of FCoV, primarily replicates in enterocytes, causing mild or asymptomatic gastrointestinal infections. In contrast, FIPV is a more virulent mutant that may lead to feline infectious peritonitis (FIP), a severe and often fatal systemic disease in a subset of infected cats [7,8]. Serologically, both FECV and FIPV can be further divided into serotypes I and II, with type I being more prevalent but remaining notoriously difficult to culture in vitro, presenting a major obstacle to experimental studies [9].

Coronavirus infection starts with the binding of its spike (S) protein to the target cell receptor, triggering the fusion of the viral and cellular membranes, which finally results in the release of the viral RNA genome into the cytoplasm [10]. Initially synthesized in an inactive prefusion form, the S protein requires a crucial cleavage process mediated by host cell protease(s) to achieve conformational activation [11]. The S protein typically experiences one or two cleavage events at specific regions known as cleavage sites (CSs). In coronaviruses with two CSs, such as SARS-CoV-2 [12] and infectious bronchitis virus [13], the first cleavage occurs at the S1/S2 junction, known as the S1/S2 CS, and is typically mediated by the host protease furin during viral biosynthesis. As demonstrated in these studies, although not strictly required for viral entry, this cleavage enhances viral infectivity and facilitates syncytium formation. Syncytium formation is the fusion of infected cells with neighboring cells, driven by viral fusion proteins such as S protein, resulting in multinucleated cells [14] that facilitate viral replication, transmission, and immune evasion, and contribute to coronavirus pathogenesis [15]. The second cleavage at the S2′ CS occurs during viral entry and is driven by host proteases as well, triggering the final conformational changes (postfusion) needed for membrane fusion and infection [16]. In contrast, coronaviruses lacking a furin-recognized S1/S2 CS, such as type II FCoV, depend solely on cleavage at the S2′ CS for activation and membrane fusion [17,18]. While the S protein cleavage pattern of type I FCoV has not yet been studied, sequence alignment with other coronaviruses suggests the presence of putative S1/S2 and S2′ CSs [9].

Type I FCoV strains account for the majority of natural infections in domestic cats (80–90%), whereas type II strains are much less common (<10%) [19]. Despite their prevalence, research on type I viruses is limited due to the lack of susceptible cell lines. Our laboratory has developed a feline intestinal epithelial cell line that supports the replication of type I FCoV strains [20], providing a valuable tool for their study. Here, we investigated three type I FCoV strains, UCD, UG-FH8, and ABA, to examine whether type I FCoV exploits intestinal protease activity to facilitate viral entry. The results will provide valuable insights into the viral activation mechanisms, host adaptation strategies, and the potential of targeting proteases for therapeutic intervention.

## Results

### Serine proteases are essential for the entry of enterocytes by FCoV

Following ingestion, FCoV encounters diverse endogenous and exogenous proteases in the intestinal tract. To investigate their roles in viral entry, we examined four major classes of mammalian intestinal proteases: serine, cysteine, metalloproteases, and aspartic proteases, using specific inhibitors in feline intestinal epithelial cells (FIEC). The cytotoxicity of each inhibitor was first assessed by 3-(4,5-dimethylthiazol-2-yl)-2,5-diphenyltetrazolium bromide (MTT) assay, and the highest concentration maintaining over 90% cell viability was selected for further experiments. The FIECs were first pre-treated with neuraminidase (NA) to enhance viral infection, as previously described [21]. The cells were then pre-treated with different concentrations of inhibitors at 37 °C for 2 h followed by inoculation of three type I FCoV strains UCD, UG-FH8 and ABA at an MOI of 0.05 in the presence of inhibitors at 37 °C for 1 h. The unbound viruses were removed by three washing steps. The FIECs were further incubated in the presence of inhibitors at 37 °C for 12 h.

**Fig 1:**
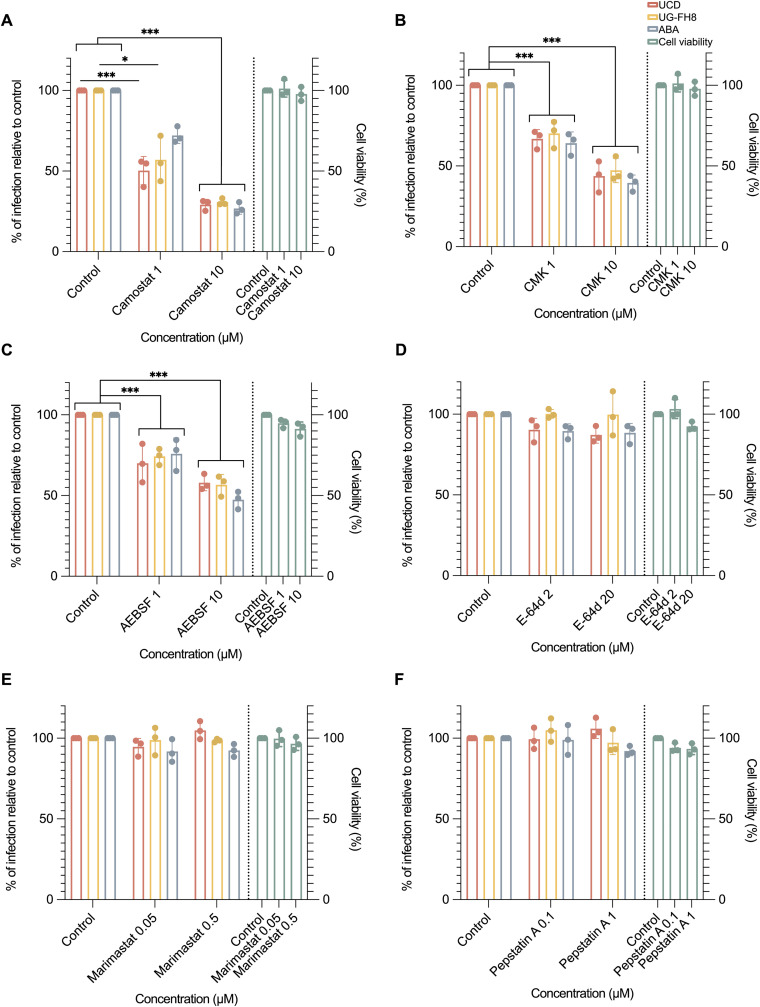
Effects of serine, cysteine, metalloproteinase, and aspartic protease inhibitors on FIEC viability and FCoV replication in FIECs. The cytotoxicity of each protease inhibitor on feline intestinal epithelial cells (FIECs) was evaluated using an MTT assay (green bars). FIECs were first pre-treated with neuraminidase for 1 hour, followed by a 2-hour incubation with the respective inhibitors. The cells were then inoculated with type I FCoV strains (UCD, UG-FH8, and ABA; MOI = 0.05) for 1 hour in the continued presence of the inhibitors. At 12 hours post-infection (hpi), cells were fixed and stained with an anti-nucleocapsid antibody to reveal infected cells. The relative infection rate was calculated by normalizing the infection rate (infected cells/total cells) to that of the untreated control. Error bars represent the standard error of the mean (SEM), n = 3. Statistical significance was determined by two-way ANOVA, except for camostat, which failed the normality test and was analyzed using the Kruskal–Wallis test. *, **, and ***, indicate p-values < 0.05, 0.01, and 0.001 respectively. Panels (A–F) show the effects of specific protease inhibitors on the replication of type I FCoV strains: **(A–C)** Serine protease inhibitors AEBSF (F_(2, 18)_ = 119.773, p < 0.001), CMK (F_(2, 18)_ = 201.437; p < 0.001) and camostat (F_(2, 18)_ = 310.521; p < 0.001); **(D)** Cysteine protease inhibitor E-64d (F_(2, 18)_ = 2.558; p = 0.074); **(E)** Metalloproteinase inhibitor marimastat (F_(2, 18)_ = 2.625; p = 0.1); **(F)** Aspartic protease inhibitor pepstatin A (F_(2, 18)_ = 0.7; p = 0.510).

As shown in Fig 1, among the tested inhibitors, three serine protease inhibitors, camostat, decanoyl-RVKR-chloromethyl ketone (CMK), and 4-(2-aminoethyl)benzenesulfonyl fluoride hydrochloride (AEBSF), exhibited potent inhibitory effects on FCoV entry. Camostat demonstrated the strongest antiviral activity with the lowest cytotoxicity, leading to approximately 80% inhibition of viral replication at a concentration of 10 μM (Fig 1A). CMK also exhibited a significant inhibition, reducing viral replication by more than 60% (Fig 1B), and AEBSF (Fig 1C) achieved 50% inhibition. In contrast, cysteine protease inhibitor (2S,3S)-trans-Epoxysuccinyl-L-leucylamido-3-methylbutane ethyl ester (E-64d) had only a minor impact on viral entry (Fig 1D). Inhibitors targeting metalloproteases and aspartic proteases showed no effect on FCoV entry (Fig 1E and 1F). These findings underscore the critical role of serine proteases in FCoV entry.

### FCoV depends on an acidic pH for enterocyte infection

Current virological models propose two distinct coronavirus entry mechanisms: the serine protease-mediated cell surface pathway or the cathepsin-mediated endosomal entry pathway [22]. The former is generally considered pH-independent, while the latter is low pH-dependent, as cathepsins require an acidic pH for their activity [23,24]. Given that cysteine proteases, including cathepsins, were found non-essential for viral infection in our system, we expected that a low pH was not critical for viral infection.

**Fig 2:**
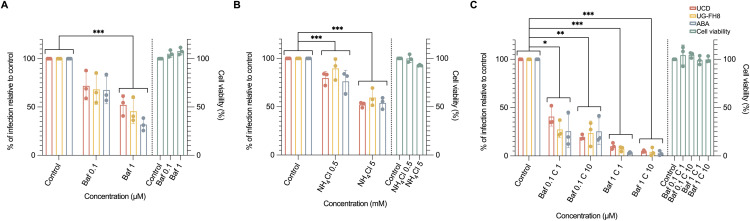
Effect of pH on the replication of FCoV. The cytotoxicity of each inhibitor on feline intestinal epithelial cells (FIECs) was assessed using an MTT assay (green bars). FIECs were first pre-treated with neuraminidase for 1 hour, followed by a 2-hour incubation with acidification inhibitors. The cells were then inoculated with type I FCoV strains (UCD, UG-FH8, and ABA; MOI = 0.05) for 1 hour in the presence of the inhibitors and maintained in inhibitor-containing medium thereafter. At 12 hours post-infection (hpi), cells were fixed and stained with an anti-viral nucleocapsid antibody to reveal the infected cells. The relative infection rate was calculated by normalizing the infection rate (infected cells/total cells) to that of the untreated control. Error bars represent the standard error of the mean (SEM), n = 3. Statistical significance was determined by two-way ANOVA, except for bafilomycin A1(Baf) and combined treatment of Baf with camostat, which failed the normality test and was analyzed using the Kruskal–Wallis test. *, **, and ***, indicate p-values < 0.05, 0.01, and 0.001 respectively. **(A)** Effect of NH₄Cl (F_(2, 18)_ = 96.783; p < 0.001) on the replication of type I FCoV strains. **(B)** Effect of Baf (F_(2, 18)_ = 67.061; p < 0.001) on the replication of type I FCoV strains. **(C)** Effect of combined treatment with Baf and camostat (F_(4, 30)_ = 239.767; p < 0.001) on the replication of type I FCoV strains. C: camostat.

To test the pH dependency, two acidification inhibitors were used: bafilomycin A1(Baf) and NH₄Cl. NA pre-treated FIEC monolayers were treated with these inhibitors for 2 hours at 37°C, followed by virus inoculation at an MOI of 0.05 in the presence of the inhibitors for 1 hour at 37°C. After infection, unbound viruses were removed by three washings with PBS, and the FIECs were further incubated with the inhibitors for an additional 12 hours at 37°C. In contrast to our expectation, as shown in Fig 2, both inhibitors significantly reduced viral infection. Specifically, 1 μM Baf inhibited infection by approximately 50% (Fig 2A), and 5 mM NH₄Cl produced a similarly significant but less pronounced inhibitory effect (Fig 2B). These results suggest that, while serine proteases are essential for viral infection, acidic pH also plays an important role in facilitating viral infection, despite the common assumption that the serine protease-mediated cell surface pathway is pH-independent.

To evaluate whether the combination of camostat and Baf can effectively block viral infection, cells were treated with both inhibitors simultaneously. Although the infection was not completely abolished, it was significantly reduced to less than 5% when treated with a combination of 10 μM camostat and 1 μM Baf (Fig 2C). These results suggest that a combined treatment with Baf and camostat suppresses viral infection more effectively than either agent alone.

### Intestinal serine proteases enhance FCoV enterocyte infection

**Fig 3:**
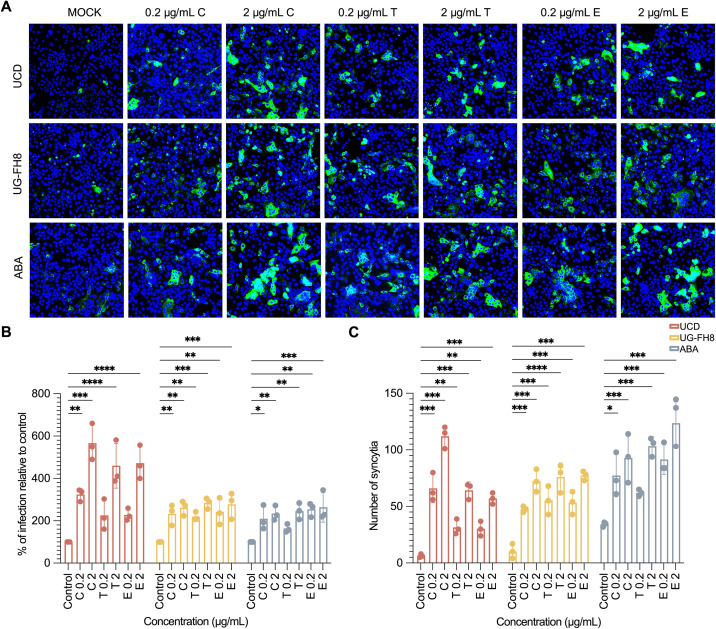
Impact of pancreatic serine proteases on FCoV replication. Feline intestinal epithelial cells (FIECs) were pre-treated with neuraminidase for 1 hour prior to inoculation with type I FCoV strains (UCD, UG-FH8, and ABA). Virus adsorption was performed in the presence of pancreatic serine proteases for 1 hour, after which cells were maintained under the same treatment conditions. At 12 hours post-infection (hpi), cells were fixed and stained with anti-FCoV nucleocapsid antibody (10A12). **(A)** Representative immunofluorescence images showing enhanced viral infection and syncytia formation following treatment with serine proteases. **(B)** Quantification of serine protease-mediated enhancement of viral infection. The relative infection rate was calculated as the number of infected cells divided by total cell number, normalized to the untreated control. Statistical significance was determined by one-way ANOVA (F_(6, 42)_ = 27.296; p < 0.001) followed by Dunnett’s post hoc test to compare treated groups with the untreated control. *, **, and *** indicate p-values < 0.05, 0.01, and 0.001, respectively. **(C)** Quantification of syncytia formation following protease treatment. Syncytia were counted from three replicate experiments and normalized to the untreated control. C: chymotrypsin; T: trypsin; E: elastase. Error bars represent the standard error of the mean (SEM), n = 3. Due to interaction effects between virus strains and inhibitor concentrations, data were analyzed separately for each strain. Statistical significance was determined by one-way ANOVA (F_(6, 42)_ = 52.687; p < 0.001) followed by Dunnett’s post hoc test to compare treated groups with the untreated control. *, **, and *** indicate p-values < 0.05, 0.01, and 0.001, respectively.

The potent inhibitory effect of serine protease inhibitors on viral infection underscores the critical role of serine proteases in this process. To investigate whether serine proteases that are naturally present in the intestines may influence viral infection, three principal intestinal serine proteases (trypsin, chymotrypsin, and elastase) [25] were tested on their impact on infection. NA-pretreated FIECs were incubated with virus in the presence of different concentrations of trypsin, chymotrypsin, or elastase for 1 hour at 37 °C. After three washing steps to remove unbound viruses, cells were further incubated for 12 hours at 37 °C. As shown in Fig 3, the addition of exogenous serine proteases not only enhanced viral infection but also significantly promoted syncytia formation. Among the viral strains tested, UCD exhibited the greatest susceptibility to serine protease-mediated enhancement, with the infectivity increasing more than fivefold compared to the mock-treated group. In contrast, UG-FH8 and ABA displayed a more moderate, twofold increase in infectivity following serine protease treatment. Additionally, ABA strain showed the strongest ability to trigger cell–cell fusion, leading to the formation of large multinucleated syncytia when serine proteases were present. These findings demonstrate that the tested intestinal serine proteases, including trypsin, chymotrypsin, and elastase, play a significant role in facilitating viral infection.

### Intestinal serine proteases cleave the FCoV S protein

**Fig 4:**
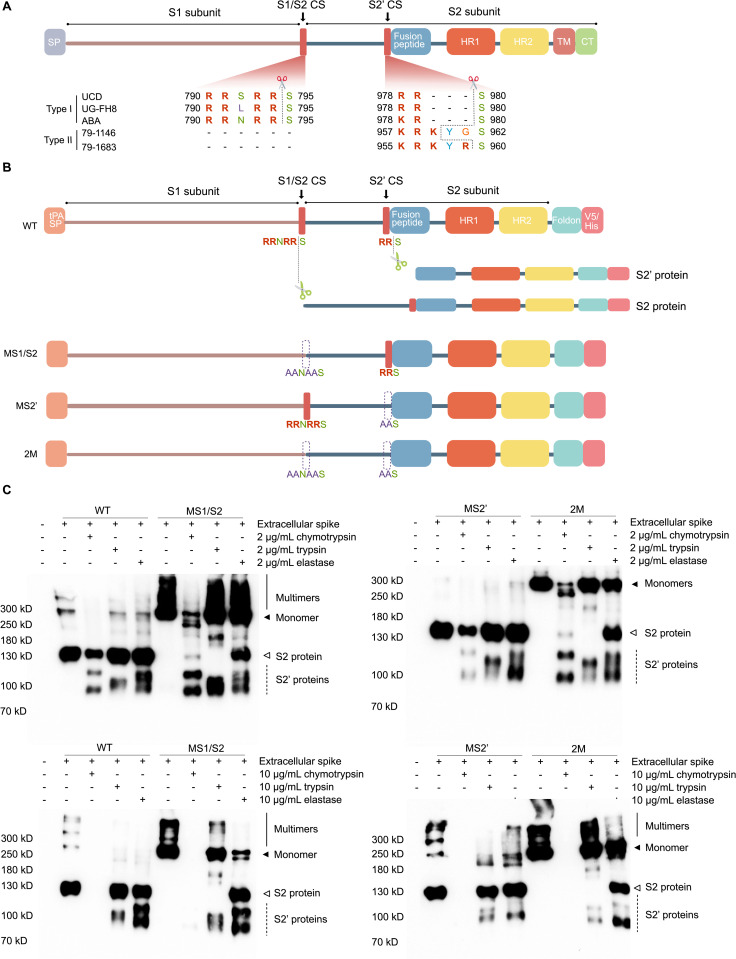
Cleavage of the FCoV spike protein by serine proteases. **(A)** Schematic of the feline coronavirus spike protein and its putative cleavage sites. Multiple sequence alignment of coronavirus spike proteins was performed using MEGA12 with the MUSCLE algorithm. Predicted protease cleavage motifs are highlighted in bold red, with scissor icons marking the predicted cleavage positions. CS: cleavage site; SP: signal peptide; HR1: heptad repeat 1; HR2: heptad repeat 2; TM: transmembrane domain; CT: cytoplasmic tail. **(B)** Schematic of the wild-type and mutant spike constructs derived from the type I FCoV strain ABA. The native transmembrane and cytoplasmic regions were replaced with a foldon trimerization domain and a C-terminal V5/His tag. Cleavage at the S1/S2 site generates the S2 protein, and cleavage at the S2′ site yields the S2′ protein. WT: wild-type spike; MS1/S2: mutant at the S1/S2 site (RRNRRS → AANAAS); MS2′: mutant at the S2′ site (RRS → AAS); 2M: double mutant at both sites. tPA SP: tissue plasminogen activator signal peptide. **(C)** Western blot analysis of spike cleavage in supernatants following serine protease treatment. HEK293T cells were transfected with spike constructs and cultured in serum-free medium for 48 h. Supernatants were collected and incubated with indicated concentrations of serine proteases at 37 °C for 30 min. Cleavage products were detected using anti-V5 western blotting. Solid line: spike multimers; solid arrow: monomeric spike; open arrow: S2 protein (from S1/S2 cleavage); dashed lines: S2′ proteins (from S2′ or alternative cleavages).

Previous studies [9,18] predicted that type I FCoV strains have two CSs in the S protein, S1/S2 and S2′, while type II strains have only the S2′ CS. In this study, we performed a sequence alignment using the strains analyzed here (UCD, UG-FH8, and ABA for type I; 79–1146 and 79–1683 for type II). Guided by the previous studies, we identified the S1/S2 CS (^790^RRNRRS^795^) at the junction between the S1 and S2 subunits, which forms a typical furin recognition motif, and the S2′ site (^978^RRS^980^) within the S2 subunit in the type I strains (Fig 4A). In contrast, the type II strains contain only the S2′ CS.

To investigate the mechanism underlying the enhanced infection mediated by intestinal serine proteases, we generated an expression construct encoding the S protein of the ABA strain, which exhibits strong syncytium-inducing activity. As illustrated in Fig 4B, to enhance the S protein secretion in HEK293T cells, the native signal peptide was replaced by the tissue plasminogen activator signal peptide (tPA-SP) [26]. The C-terminus was fused with the trimerization domain of bacteriophage T4 fibritin foldon to promote its trimerization [27], and the transmembrane and cytoplasmic domain of the S protein were replaced by a V5/6x His tag to allow the detection of cleaved products using an anti-V5 antibody. To identify the specific CSs recognized by these serine proteases, mutations were introduced into these sites (Fig 4B): in plasmid MS1/S2, the S1/S2 CS was mutated (^790^RRNRRS^795^ → ^790^AANAAS^795^); in plasmid MS2′, the S2′ CS was mutated (^978^RRS^980^ → ^978^AAS^980^); in plasmid 2M, both CSs were mutated. Cleavage at the S1/S2 CS produces the S2 protein, and S2′ cleavage generates the S2′ protein.

HEK293T cells were seeded in 24-well plates and transfected with the wild-type ABA spike or the mutated constructs when they reached 70% confluence. After 48 hours of transfection, culture supernatants were collected, and the cells were lysed by ultrasonication followed by centrifugation to obtain the intracellular spike. Both the supernatants and cell lysates were then treated with serine proteases (2 μg/mL or 10 μg/mL) at 37 °C for 30 min. The cleavage profiles of intracellular spike proteins obtained from cell lysates are shown in S1 Fig, and extracellular spike proteins secreted into the supernatant are shown in Fig 4C.

In HEK293T cells, the wild-type ABA S protein was detected in three major forms: (1) high-molecular-weight multimeric bands (>300 kD) (solid line), corresponding to assembled spike complexes; (2) a 280 kD monomer (solid arrowhead), corresponding to the full-length, uncleaved S protein; and (3) a 130 kD fragment called S2 protein (open arrowhead), representing the S2 subunit generated by endogenous furin-mediated cleavage at the S1/S2 boundary. The multimer and monomer were primarily detected intracellularly (S1 Fig), whereas the cleaved S2 protein was predominantly released into the supernatant (Fig 4C). The immature S protein retained intracellularly was more susceptible to non-specific cleavage by serine proteases, resulting in multiple heterogeneous cleavage products without clearly defined or nameable fragments (S1 Fig). In contrast, the mature S protein released into the supernatant was more resistant to cleavage (Fig 4C). Since the mature spike in the supernatant undergoes proper post-translational modifications, such as trimerization, N-glycosylation, and palmitoylation [28], it better represents the true cleavage scenario. Therefore, we will focus on the cleavage of the mature S protein released into the supernatant, as shown in Fig 4C.

#### Cleavage profile of wild-type FCoV S protein.

As expected, trypsin cleaved at the canonical S2′ site (^978^RRS^980^), producing a distinct 110 kD S2′ protein (Fig 4C, dashed line).

At a concentration of 2 μg/mL, chymotrypsin cleaved the S2 subunit at two distinct sites, producing two S2′ protein fragments: a 120 kD band and a 90 kD band (dashed lines). These sites, located upstream and downstream of the canonical S2′ CS, were designated S2′a CS (upstream) and S2′b CS (downstream), with the resulting proteins named S2′a protein and S2′protein, respectively. At an increased concentration of 10 μg/mL, chymotrypsin induced over-cleavage, resulting in no fragments detectable.

Similar to chymotrypsin, elastase at 2 μg/mL cleaved the S2 subunit at the S2′a and S2′b CS, generating smeared 120 kD (S2′a) and 90 kD (S2′b) bands. At 10 μg/mL, elastase cleavage became more distinct, producing well-defined S2′a and S2′b proteins, suggesting that higher elastase concentrations are required for complete cleavage.

#### Cleavage profile of MS1/S2 mutant.

In the MS1/S2 mutant, disruption of the furin CS abolished furin-mediated production of the S2 protein, so only the uncleaved, full-length S protein was detected. However, treatment with chymotrypsin or elastase restored the generation of the S2 fragment, indicating that these proteases cleaved sequences adjacent to but distinct from the canonical furin CS. Thus, mutation of the S1/S2 site did not affect the susceptibility to chymotrypsin- or elastase-mediated cleavage.

In contrast, trypsin failed to cleave the mutated S1/S2 site, and no S2 protein was detected, suggesting that trypsin targets the same motif as furin. Interestingly, cleavage by trypsin at the downstream S2′ site was also altered in this mutant, resulting in two smeared and partially overlapping bands: a ~ 115 kD fragment (designated S2′-alt1 protein) and a ~ 90 kD fragment (designated S2′-alt2 protein). These results suggest that mutation of the S1/S2 site indirectly alters trypsin activity at the S2′ region, possibly due to conformational changes in the S protein that affect downstream cleavage site accessibility.

#### Cleavage profile of MS2′ mutant.

In the MS2′ mutant, chymotrypsin cleavage remained largely unaffected, generating both the 120 kD (S2′a) and 90 kD (S2′b) proteins, consistent with the wild-type cleavage profile. In contrast, elastase predominantly produced the 90 kD S2′b fragment, while generation of the 120 kD S2′a protein was markedly reduced, suggesting that the S2′ mutation selectively impairs cleavage at the S2′a site.

Trypsin cleavage in the MS2′ mutant also deviated from the wild-type pattern. Instead of the canonical 110 kD S2′ fragment, trypsin produced a ~ 115 kD band (designated S2′-alt1) and a ~ 90 kD band (S2′-alt2), a similar cleavage pattern observed in the MS1/S2 mutant. At 2 μg/mL trypsin, S2′-alt1 was the predominant fragment, with only a faint smear corresponding to S2′-alt2. At 10 μg/mL, both fragments were clearly detectable at comparable levels. These findings indicate that, in the absence of a functional S2′ site, trypsin re-targets its cleavage activity to alternative regions flanking the original S2′ motif, thereby generating compensatory cleavage products (S2′-alt1 and S2′-alt2). This observation underscores the critical role of the S2′ site in spike activation and further the existence of compensatory cleavage mechanisms. Given that coronaviruses are RNA viruses with high mutation rates, such redundancy in spike cleavage sites may provide an evolutionary advantage by preserving spike activation despite genetic variability.

#### Cleavage profile of 2M mutant.

The cleavage profile was similar to that of the MS2′ mutant, except that trypsin failed to produce the S2 protein.

Based on a multiple sequence alignment of spike proteins from three FCoV strains (S2 Fig), two conserved motifs were identified as potential alternative cleavage sites. The first is ^941^RS^942^ (S2′-alt1), which conforms to a typical R ↓ S spike cleavage pattern. The second is ^1104^KKC^1106^ (S2′-alt2), which contains a dibasic KK motif; the presence of two lysines could enhance substrate binding to the negatively charged S1 pocket of serine proteases. These conserved features suggest that both regions may function as alternative cleavage sites.

### Spike cleavage at the alternative S2′ site retains fusion ability but shows a substantial reduction in efficiency

**Fig 5:**
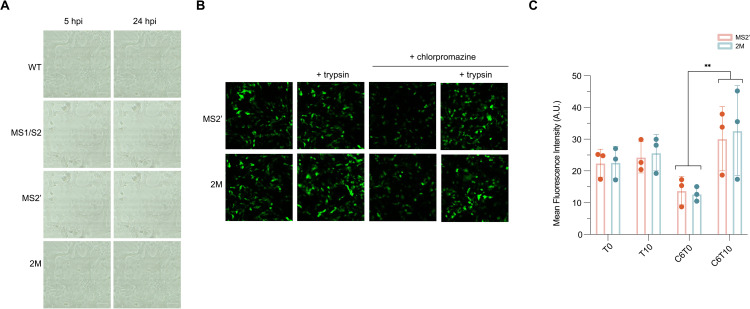
Entry of pseudotyped viruses carrying wild-type or mutant FCoV Spike. **(A)** Optical microscopical images showing cytopathic effects induced by four Spike-pseudotyped lentiviruses (wild-type, MS1/S2, MS2’, and 2M). Neuraminidase (NA)-pretreated FIEC monolayers were inoculated with each pseudotyped virus, and viral entry–associated CPE was documented at 5 hpi and 24 hpi. **(B)** Representative fluorescence microscopy images of FIEC monolayers at 48 hpi following inoculation with MS2’ or 2M Spike-pseudotyped lentiviruses. Cells were pretreated with NA and infected under the following conditions: untreated, supplemented with trypsin (10 µg/mL), treated with chlorpromazine (CPZ, 6 µM), or treated with both CPZ and trypsin. **(C)** Quantitative analysis of viral entry for MS2’ and 2M pseudoviruses under the treatment conditions described in panel (B). Data represent the mean EGFP fluorescence intensity measured from 10 random fields per condition. Statistical significance was determined by two-way ANOVA followed by Tukey HSD post hoc test. F_(3, 16)_ = 6.796, p = 0.004.** indicates p < 0.01. T0: without trypsin; T10: 10 µg/mL trypsin; C6: 6 µM CPZ.

To determine whether trypsin-mediated cleavage at the alternative sites (S2′-alt1 and S2′-alt2) could support viral entry, pseudotyped lentiviruses bearing wild-type or mutant FCoV spikes were generated. To verify that EGFP expression reflected authentic viral entry, pseudoviruses were first inoculated onto felis catus whole fetus (Fcwf) and HEK293T cells, both of which are non-permissive for FCoV entry. As expected, no fluorescence was detected in these cells or in FIEC monolayers without NA pretreatment, confirming that EGFP expression resulted from pseudovirus entry and delivery of the reporter gene. As shown in Fig 5A, pseudoviruses carrying wild-type or MS1/S2 spike induced extensive syncytium formation and CPE as early as 5 hours post-infection (hpi), and the monolayer was completely disrupted by 24 hpi. These results further demonstrate that cleavage at the S1/S2 CS is not essential for viral entry. In contrast, pseudoviruses bearing the MS2’ or 2M spike produced almost no detectable CPE, confirming the essential role of the canonical S2′ CS in S protein activation and entry.

Since wild-type and MS1S2 pseudoviruses disrupted the cell monolayer within 24 hours, quantitative analysis of entry was not feasible. Therefore, we focused subsequent experiments on MS2’ and 2M pseudoviruses; the results are shown in Fig 5B and 5C. Although these mutants did not cause obvious CPE, fluorescence was detectable at 48 hpi and did not significantly increase with additional trypsin treatment. We hypothesized that in the absence of a canonical S2’ CS, the virus mainly utilize an endosomal entry pathway. To test this, we ultilizes chlorpromazine (CPZ) at a safe dose of 6 μM to inhibit endosomal entry pathway. CPZ treatment reduced fluorescence, indicating involvement of clathrin-dependent endocytosis. The addition of trypsin significantly enhanced fluorescence, suggesting that cleavage at alternative sites by trypsin can generate fusion-competent peptides. However, the absence of CPE or syncytium formation following trypsin treatment indicates that peptides generated from alternative CSs have markedly reduced fusion capacity. One possible explanation is that higher trypsin concentrations are required for complete cleavage at alternative CSs, but such concentrations would compromise the cell monolayer, precluding their use in our *in vitro* experimental system.

### Natural mutations in the S2′ cleavage site modulate trypsin-mediated spike processing

Given that artificially introduced mutations at the S2′ CS alter the trypsin cleavage profile, we sought to determine whether the S2′ CS is subject to natural variation. To this end, we retrieved 167 type I FCoV S protein sequences from GenBank and performed multiple sequence alignment using the MUSCLE algorithm in MEGA (version 12) software. Sequence conservation across the S2′ region was visualized using WebLogo 3.

**Fig 6:**
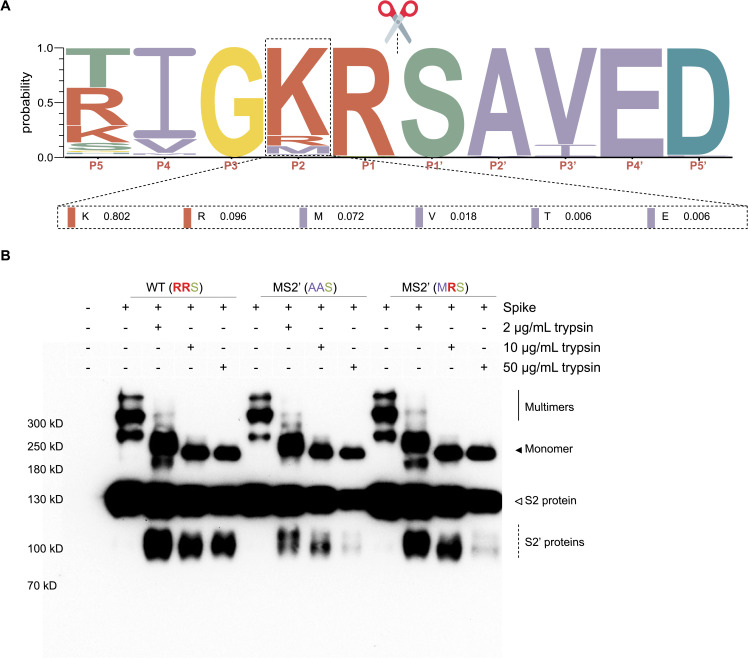
Natural variation at the S2′ cleavage site alters trypsin-mediated processing of the FCoV spike protein. **(A)** Sequence logo of the S2′ cleavage site generated from multiple sequence alignment of 167 type I FCoV spike protein sequences. The canonical basic residue at the P2 position (K/R) is replaced by non-basic amino acids (M, V, T, E) in 10.2% of isolates, with methionine (M) being the most frequent substitution (7.2%). **(B)** Trypsin-mediated cleavage profiles of three spike variants: wild-type (RRS), a non-cleavable mutant (AAS), and a naturally occurring mutant (MRS). HEK293T cells were transfected with spike constructs and cultured in serum-free medium for 48 **h.** Supernatants were collected and incubated with increasing concentrations of trypsin (2, 10, and 50 μg/mL) at 37 °C for 30 min. Cleavage products were detected using anti-V5 western blotting. Solid line: spike multimers; solid arrow: monomeric spike; open arrow: S2 protein (from S1/S2 cleavage); dashed lines: S2′ proteins (from S2′ or alternative cleavages).

As shown in Fig 6A, the canonical basic residue at the P2 position (K/R) is replaced by alternative amino acids in 10.2% of the analyzed sequences. Among these substitutions, methionine (M) is the most prevalent, appearing in 7.2% of cases, followed by valine (V), threonine (T), and glutamic acid (E). To evaluate the functional consequences of this natural variation, we focused on the MRS variant, in which the canonical RRS motif is replaced by MRS in a subset of viral isolates. Since RRS represents a canonical trypsin recognition motif, we assessed how this substitution affects trypsin-mediated cleavage.

We compared the cleavage profiles of three spike variants: wild-type (RRS), MS2’ (AAS), and the naturally occurring MRS mutant. Each variant was incubated with increasing concentrations of trypsin (2, 10, and 50 μg/mL), followed by analysis of cleavage patterns via SDS-PAGE and western blot. As shown in Fig 6B, the wild-type RRS variant consistently produced a single, stable cleavage product across all trypsin concentrations, indicating specific processing at the canonical site. In contrast, the AAS variant exhibited alternative cleavage bands at all concentrations, producing S2′-alt1 and S2′-alt2 fragments, indicative of non-canonical cleavage. Interestingly, the MRS variant initially displayed an RRS-like cleavage profile at 2 μg/mL trypsin. However, upon exposure to 10 and 50 μg/mL trypsin, its cleavage pattern shifted and closely resembled that of the AAS mutant, producing S2′-alt1 and S2′-alt2 fragments. These results suggest that although the MRS variant retains one arginine residue, its recognition by trypsin is destabilized at higher enzyme concentrations, resulting in a cleavage shift.

Together, these findings reveal that the S2′ CS in FCoV is subject to natural mutation, and such variation compromises the stability of trypsin recognition. Importantly, the S protein remains cleavable despite changes in both the S2′ sequence and protease concentration, highlighting the inherent flexibility of the S2′ activation mechanism.

### The feline colonic contents and feces share similar serine protease profiles

To confirm the presence of intestinal serine proteases, three colonic fecal samples collected from deceased cats (cat 1–3) were prepared as 10% and 1% (w/v) suspensions and analyzed using gelatin-embedded zymography. For comparison, three fecal samples collected from shelter cats (cat 4–6) were also prepared as 10% suspensions and included in the analysis.

**Fig 7:**
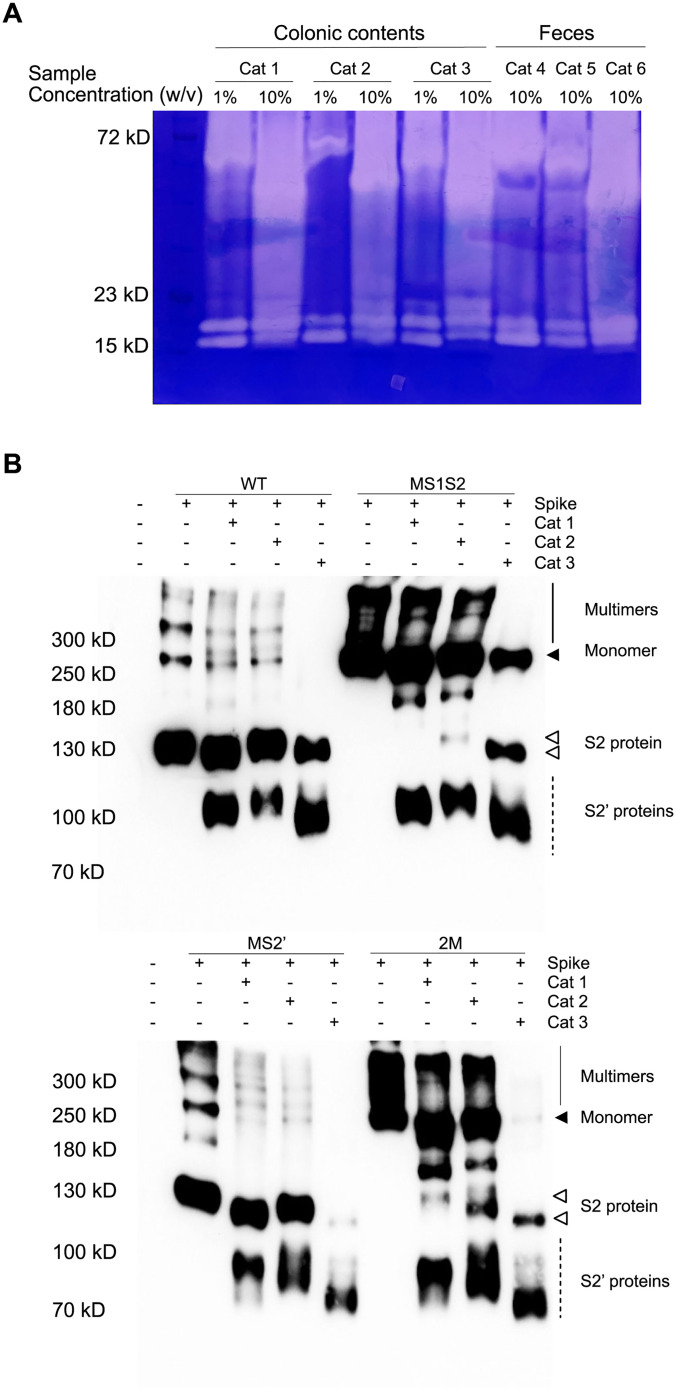
The identification and cleavage of feline fecal serine proteases. **(A)** Fecal serine protease zymography. Colonic fecal samples were collected and prepared as 1% and 10% suspensions before zymographic analysis. For comparison, three additional fecal samples from healthy shelter cats were collected and prepared as 10% suspensions. Transparent bands indicate the presence of serine proteases. **(B)** Cleavage of feline fecal serine proteases. Supernatants containing secreted spike protein from spike-transfected HEK293T cells were collected and incubated with 10% colonic fecal suspensions at 37°C for 30 minutes. Cleaved products were analyzed by western blotting using an anti-V5 antibody. Solid line: spike multimers; solid arrow: monomeric spike; open arrow: S2 protein (from S1/S2 cleavage); dashed lines: S2′ proteins (from S2′ or alternative cleavages).

As shown in Fig 7A, the samples generally exhibited a consistent pattern of serine protease activity, revealing two distinct groups of serine proteases. The first group was detected as a strong ~72 kD band when samples were prepared as 1% suspensions. Interestingly, increasing the sample concentration to 10% caused this band to migrate to a lower position on the gel, with slight variation between samples. The second group consisted of bands ranging from 15–20 kD. In colonic samples, three distinct bands were consistently observed, whereas only two were seen in fecal samples. This difference suggests that one protease may be lost, degraded, or inactivated during transit through the intestine or after excretion.

As the fecal sample from Cat 2 displayed distinct protein bands, these bands were excised and subjected to mass spectrometry analysis. The resulting protein identifications are summarized in Table 1. Notably, most proteases yielded only one or two peptides in the mass spectrometry analysis. This is likely because the proteins remained in their native, non-denatured conformations, which limited the accessibility of cleavage sites for trypsin digestion. Since mass spectrometry requires proteins to be enzymatically digested by trypsin into smaller peptides for identification, incomplete digestion can result in very few detectable peptides, thereby reducing identification confidence. The analysis of the 15–20 kD protein group resulted in the identification of two feline serine proteases: trypsin and chymotrypsin-like elastase family member 1 (CELA1). Surprisingly, chymotrypsin was not detected. To validate these findings, the 15–20 kD bands from cat 3 was also analyzed by LC-MS/MS, yielding the same result: only trypsin and CELA1 were detected, with no evidence of chymotrypsin. In another feline fecal proteome study from 10 healthy cats, chymotrypsin-like elastase family member 3B and kallikrein-1 were the only two serine proteases detected [29]. Therefore, chymotrypsin may undergo autolysis or be degraded by other proteases during its passage through the intestine. In addition to feline serine proteases, few bacterial serine proteases were also detected such as prolyl oligopeptidase. Although these bacterial serine proteases have molecular weights far above 20 kD, they were detected at ~20 kD likely due to proteolytic fragmentation during sample handling or endogenous activity. In contrast, analysis of the 72 kD band did not identify any definitive serine protease candidates. One possibility is bacterial oligopeptidase B from a *Flavobacteriaceae* species; however, this enzyme is known to preferentially hydrolyze short peptides (typically fewer than 30 residues) [30]. Given that the zymography substrate used was gelatin, a large, denatured protein, this enzyme is unlikely to account for the observed proteolytic activity. Therefore, the 72 kD band may correspond to a previously uncharacterized bacterial serine protease that is not currently annotated in available protein databases.

**Table 1:** Serine proteases identified in feline colonic fecal samples by LC-MS/MS.

Protein Name	Organism Species	Peptides	% Coverage	MW (kD)	Uniprot
**15-20 kD band**					
**Chymotrypsin-like elastase family member 1**	*Felis catus*	2	11.4	27.63	A0A5F5XS71
**Trypsin**	*Felis catus*	2	4	27.42	A0A337S8S6
**Prolyl oligopeptidase**	*Bradyrhizobium yuanmingense*	2	3.8	76.19	A0AAW4SBP3
**S8 family peptidase**	*Streptomyces sp. ISL-94*	1	5.4	41.47	A0A944PSA5
**Trypsin-like serine protease**	*Myxococcales bacterium*	1	5	53.18	A0A9D7H5R3
**72 kD band**					
**Oligopeptidase B**	*Flavobacteriaceae bacterium*	1	1.2	80.86	A0A2E1EQG2
**LD-carboxypeptidase**	*Mesohalobacter halotolerans*	1	3	33.21	A0A4V6XY98
**Rhomboid family intramembrane serine protease**	*Streptomyces tateyamensis*	1	3.4	26.26	A0A2V4N1D6

### Cleavage of FCoV S protein by feline colonic contents

To assess S protein cleavage by endogenous intestinal serine proteases, the FBS-free supernatant from spike-transfected HEK293T cells was collected and incubated with three colonic content samples (1% w/v) for 30 min at 37°C. The samples were then mixed with 5x non-reducing loading buffer, boiled for 5 min, and analyzed by SDS-PAGE followed by western blotting using an anti-V5 antibody. As shown in Fig 7B, the cleavage pattern observed in the colonic suspension from Cat 1 closely resembled that of trypsin. It failed to cleave the mutated S1/S2 site and produced a single, defined S2′ band. Upon S2′ mutation, the cleavage pattern shifted to a ~ 115 kD fragment and a smeared 90 kD band, mimicking the pattern seen with 2 μg/mL trypsin (Fig 4C).

In contrast, the fecal sample from Cat 2 displayed a more adaptable cleavage profile. When the S2′ site was intact, cleavage produced a single, well-defined band consistent with trypsin-mediated processing. However, upon mutation of the S2′ site, the cleavage pattern shifted to two bands characteristic of elastase-like activity. This shift suggests that, in the absence of a functional trypsin cleavage site, elastase can replace trypsin as the predominant protease driving spike processing.

Cat 3 consistently displayed an elastase-like cleavage pattern. With an intact S2′ site, it produced a smeared ~120 kD band and a distinct 90 kD fragment. Upon S2′ mutation, the 90 kD fragment became predominant. Notably, a chymotrypsin-like pattern was not observed in any of the three samples, consistent with mass spectrometry results that identified trypsin and CELA1, but not chymotrypsin, in the colonic contents.

Interestingly, treatment of the S protein with these colonic content samples resulted in differential migration shifts, especially in the S2 and S2’ subunit, which migrated faster than untreated spike. This shift is likely due to the presence of high levels of sialidases in these samples, which remove terminal sialic acids from N- or O-glycosylated S proteins, thereby causing a migration shift [28,31].

### The role of membrane-bound serine proteases in the replication of FCoV in enterocytes

Given that the intestinal mucus layer impedes pancreatic proteases from accessing epithelial cells [32,33], potentially limiting their direct influence on FCoV entry into these cells, we explored whether membrane-bound serine proteases could facilitate FCoV infection of enterocytes. Human TMPRSS2 is widely recognized for its critical role in SARS-CoV-2 infection [34], and recent research by Mettelman et al. demonstrated that feline TMPRSS2 enhances replication of the type I FIPV Black strain in Fcwf cells [35]. Furthermore, previous observations in our laboratory suggested elevated feline TMPRSS11D expression in highly susceptible FIECs (low passage cells) compared to those with low susceptibility (high passage cells). Based on these findings, we aimed to investigate the roles of these two membrane-bound serine proteases, TMPRSS2 and TMPRSS11D, in supporting FCoV infection.

As we have not identified commercial antibodies that cross-react with feline proteins, we utilized reverse transcription (RT)-PCR to evaluate the endogenous expression profiles of TMPRSS2 and TMPRSS11D in enterocytes from feline colon tissues. The colon was chosen because it serves as a primary site of viral persistence [36,37], and the susceptible cell line FIEC used in this study is also derived from colonic tissue [20]. Total RNA was isolated from six feline colonic samples, including the FIECs. It should be noted that a subset of specimens underwent two freeze–thaw cycles during processing, resulting in variable RNA integrity. As shown in S3 Fig, the transcripts of both TMPRSS2 and TMPRSS11D were detected in most of the samples. The attenuated or negative TMPRSS11D signal in certain samples likely reflects a combination of its intrinsically low abundance and RNA degradation resulting from freeze–thaw processes. These results indicate endogenous transcription of TMPRSS2 and TMPRSS11D in feline colon and colon-derived FIECs, though further validation at the protein level is warranted.

#### The detection of membrane-bound serine proteases in stably expressing FIEC cultures.

FIECs stably expressing V5-tagged TMPRSS2 and TMPRSS11D were generated under hygromycin selection. Expression of the protein was confirmed by immunofluorescence (IF) staining and western blot using an anti-V5 antibody. As transmembrane proteins, both TMPRSS2 and TMPRSS11D exhibited positive cell surface signals in IF assays without permeabilization (S4A Fig), confirming their plasma membrane localization. Western blot analysis revealed distinct migration patterns, with TMPRSS2 detected at ~60 kD and TMPRSS11D at ~50 kD (S4B Fig). However, under the tested conditions, no proteolytically cleaved forms of TMPRSS2 or TMPRSS11D were observed, despite their cleaved forms being generally associated with enzymatic activation [38,39].

#### Membrane-bound serine proteases enhance infection and syncytia formation.

**Fig 8:**
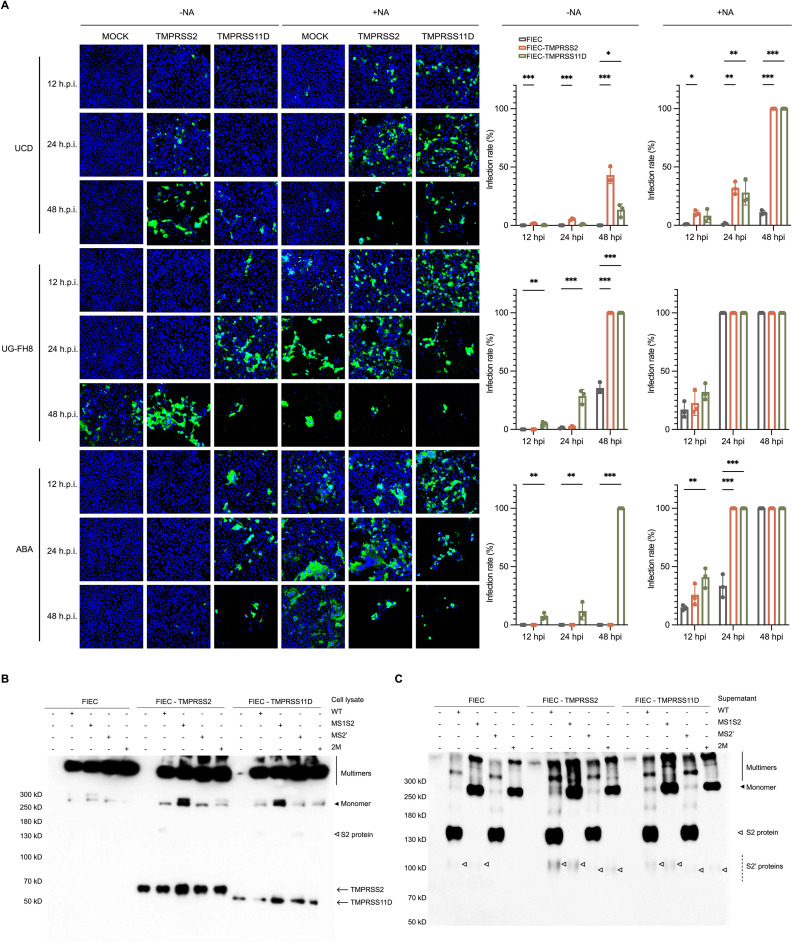
Impact of TMPRSS2 and TMPRSS11D on FCoV replication and spike cleavage. **(A)** TMPRSS2 and TMPRSS11D enhance viral infection. Feline intestinal epithelial cells (FIECs) stably expressing TMPRSS2 or TMPRSS11D were pre-treated with or without neuraminidase (NA), followed by infection with type I FCoV strains (UCD, UG-FH8, or ABA) at an MOI of 0.005. At 12, 24, and 48 hours post-infection (hpi), cells were fixed and stained with an anti-nucleocapsid antibody. Representative immunofluorescence images and quantification show increased infection rates in TMPRSS2- and TMPRSS11D-expressing cells compared to controls. Infection was quantified as the percentage of nucleocapsid-positive cells among total cells. Data represent mean ± SEM (n = 3). Statistical analysis was performed separately for each virus strain, neuraminidase treatment, and time point due to interaction effects, using one-way ANOVA F_(2, 108)_ = 576.919 with Dunnett’s post hoc test to assess differences between cell lines. *p < 0.05, **p < 0.01, ***p < 0.001. **(B, C)** Spike cleavage in FIECs expressing TMPRSS2 or TMPRSS11D. Cells were transfected with V5-tagged spike constructs, and at 48 hours post-transfection, cell lysates (B) and supernatants (C) were collected. Cleavage products were analyzed by western blot using an anti-V5 antibody. Expression of full-length and cleaved spike fragments was evaluated to determine proteolytic activity. Solid line: spike multimers; solid arrow: monomeric spike; open arrow: S2 protein (from S1/S2 cleavage); dashed lines: S2′ proteins (from S2′ or alternative cleavages).

FIECs stably expressing TMPRSS2 or TMPRSS11D were pre-treated with NA or left untreated, then infected with three type I FCoV strains (UCD, UG-FH8, and ABA) at an MOI of 0.005 for 1 h at 37 °C. After removing unbound viruses, cells were maintained in FBS-free medium and fixed at 12, 24, and 48 hpi. The results are presented in Fig 8A.

Without NA pretreatment, UCD and ABA failed to infect FIEC regardless of the inoculation duration, highlighting the necessity of NA treatment. However, UG-FH8 was an exception, displaying significant infection at 48 hpi, albeit at a much slower rate than when NA treated. Following NA pretreatment, all three strains successfully infected FIEC; UCD exhibited the lowest infection. UG-FH8 demonstrated rapid infection progression in NA-pretreated cells, causing severe CPE by 24 hpi characterized by extensive cell lysis and death, disruption of the cell monolayer, and the formation of small to medium-sized syncytia from the remaining cells. By 48 hpi, over 90% of the cells had detached due to CPE. ABA strain exhibited intermediate infectivity but was characterized by extensive syncytia formation.

In the absence of NA pretreatment, TMPRSS2 enabled UCD infection, which remained limited at 12 hpi but showed extensive infection and increased syncytia formation by 48 hpi For UG-FH8, TMPRSS2 had no significant effect at 12 and 24 hpi compared to the mock group but promoted infection at 48 hpi, as indicated by fewer remaining cells. ABA infection at 12 and 24 hpi was not detected, and only minimal infection was observed at 48 hpi In contrast, TMPRSS11D had a minor effect on UCD infection but markedly enhanced UG-FH8 and ABA infection. By 48 hpi, nearly all cells infected with UG-FH8 and ABA had undergone complete detachment due to CPE. In conclusion, in the absence of NA pretreatment, TMPRSS2 fully enabled UCD infection, significantly enhanced UG-FH8 infection, and slightly promoted ABA infection. TMPRSS11D had a minor effect on UCD but strongly facilitated ABA infection and markedly enhanced UG-FH8 infection.

In the NA-pretreated group, TMPRSS2 and TMPRSS11D exhibited similar infection-enhancing effects on all three strains, leading to increased infection rates and enhanced syncytia formation. These findings indicate that TMPRSS2 and TMPRSS11D play crucial roles in FCoV infection, with TMPRSS2 primarily facilitating UCD infection, while UG-FH8 and ABA infections are predominantly promoted by TMPRSS11D.

#### Cleavage of spike by membrane-bound serine proteases.

To determine whether TMPRSS2 and TMPRSS11D process the S protein, FIECs stably expressing either protease were transfected with spike-expressing constructs; unmodified FIECs served as controls. Cleavage products were assessed by western blot. Expression of V5-tagged TMPRSS2 and TMPRSS11D was confirmed in cell lysates (Fig 8B), and spike cleavage products were detected in the corresponding supernatants (Fig 8C). Notably, in native FIECs expressing the wild-type spike, a 110 kD S2′ fragment was observed even in the absence of exogenous protease treatment, indicating the presence of endogenous protease(s) capable of cleaving at the S2′ site. In contrast, HEK293T cells showed no detectable S2′ cleavage product unless treated with exogenous proteases (Fig 4C). Mutation of the S2′ cleavage site abolished the 110 kD S2′ protein in FIECs, indicating that the responsible endogenous protease(s) cleave specifically after arginine residues, consistent with trypsin-like specificity.

Both TMPRSS2 and TMPRSS11D were able to cleave the S2′ site, producing the same 110 kD fragment as trypsin. When the canonical S2′ site was mutated, an alternative cleavage product was observed in cells expressing TMPRSS2 or TMPRSS11D. This new fragment migrated faster than the wild-type S2′ subunit, suggesting cleavage at a downstream site with a lower molecular weight. These results suggest that TMPRSS2 and TMPRSS11D can utilize a downstream alternative CS when the canonical S2′ site is disrupted. Similar to the compensatory cleavage observed with trypsin, this flexibility highlights the critical role of the S2′ region in spike activation and reflects the virus’s evolutionary adaptation to preserve this essential entry step.

## Discussion

Over vast evolutionary timescales, viruses have evolved sophisticated mechanisms to exploit host cell biology, optimizing replication and transmission [40]. As the initial site of infection, the feline intestinal tract represents one of the most dynamic and complex systems within the host, making it crucial to understand how gastrointestinal environmental factors influence FCoV entry and replication. The intestinal mucosa is a site of abundant proteolytic activity, continuously exposed to a diverse array of proteases originating from both host and luminal microbiota [2]. This protease-rich environment likely facilitates coronavirus entry, given that S protein proteolytic cleavage initiates viral infection [41]. Using specific inhibitors, we examined the role of different protease families in viral entry. Although previous studies have shown that metalloproteases and cysteine proteases, particularly cathepsins B and L, can activate the S protein of FCoV and other coronaviruses [42–45], their inhibition minimally affected the entry of the FCoV strains tested in this study (UCD, UG-FH8, and ABA). In contrast, serine protease inhibitors significantly reduced FCoV infection, indicating that viral entry relies primarily on serine protease activity. However, even at a high but non-toxic concentration of 100 μM camostat, inhibition plateaued, suggesting that blocking serine proteases alone is not sufficient for complete inhibition. Thus, in the absence of serine protease activity, other proteases partially compensate to sustain infection albeit with markedly reduced efficiency. In summary, our findings indicate that FCoV exploits the protease-rich intestinal environment, with serine proteases playing a central role in viral entry.

It is plausible that FCoV predominantly relies on serine proteases for S protein activation, given their high abundance in the intestinal environment [46]. In this study, chymotrypsin, trypsin, and elastase, three major pancreatic serine proteases present in intestines, were shown to facilitate FCoV infection and syncytia formation, suggesting that FCoV exploits these locally abundant enzymes to activate its S protein. This adaptation likely reflects an evolutionary strategy to leverage the intestinal milieu. Notably, serine proteases differ in substrate specificity, leading to S2′ fragments of different sizes. This reflects the conformational flexibility of the spike, which exposes multiple accessible sites. A marked distinction is observed between immature (S1 Fig) and mature S proteins (Fig 4C), which display fundamentally different susceptibilities to proteolytic cleavage. In immature spikes, the full exposure of CSs permits indiscriminate digestion by serine proteases, resulting in nonfunctional fragments and loss of structural integrity. In contrast, mature S proteins undergo conformational changes that sterically shield nonproductive CSs while selectively exposing regions that yield functional S2′ fragments. This regulated processing ensures the generation of fusion-competent spike products while preventing destructive over-cleavage. These findings further illustrate FCoV’s adaptation to the intestinal environment, where spike maturation serves to protect against excessive proteolysis by abundant host proteases.

Unlike pancreatic serine proteases, which are compartmentalized by the intestinal mucosal barrier [47], membrane-bound serine proteases offer a more accessible and consistent means of facilitating viral entry. We found that TMPRSS2 and TMPRSS11D significantly enhance FCoV replication in FIECs and notably enable the UCD and ABA strains to overcome the sialic acid barrier and infect cells that otherwise remain non-permissive without sialidase pretreatment, showing potent role of these proteases in promoting viral infection. Notably, the S2′ cleavage occurred independently of S1/S2 processing (as observed in the MS1S2 mutant), resembling the activation mechanism observed in type II FCoV strains, which lack a canonical S1/S2 CS yet can be activated through direct S2′ cleavage [17]. In contrast, for SARS-CoV-2, which also possesses a functional furin CS (P**RR**A**R**) at S1/S2, cleavage at the S2′ site can only occur after the S1/S2 site has been cleaved or the receptor ACE2 is engaged [48,49].

In summary, S1/S2 processing is not essential for FCoV entry. As a result, when this site is mutated, the virus does not appear to have evolved a compensatory mechanism to process it, and such mutations do not substantially affect infectivity. In contrast, the S2′ site is functionally indispensable and to ensure successful spike activation, FCoV has evolved additional cleavage motifs in the vicinity of the S2′ CS, enabling activation even when the primary S2′ motif is disrupted. This compensatory CSs highlight the critical role of S2′-mediated cleavage in viral entry and underscores the evolutionary adaptability of coronaviruses in preserving infectivity under selective pressure or genetic variation.

Although serine protease-mediated viral entry at the cell surface is typically considered pH-independent [50], our results indicate that FCoV infection paradoxically requires both serine protease activity and low pH. This suggests a hybrid entry mechanism involving both the cell surface and endosomal pathways. In the cell surface route, FCoV employs serine proteases to cleave and prime the S protein. However, unlike classical surface fusion models where cleavage alone is sufficient, an acidic environment remains necessary to trigger membrane fusion. Similar pH dependence has been reported in SARS-CoV-2 [51], type II FCoV [17], and avian infectious bronchitis virus [52], where virions activated by serine proteases still depend on low pH for fusion. In the endosomal route, FCoV enters host cells via endocytosis. Within endosomes, serine proteases could mediate spike activation. In this context, the drop in pH within endosomes or lysosomes is essential to trigger fusion between the virus and the membranes.

**Fig 9:**
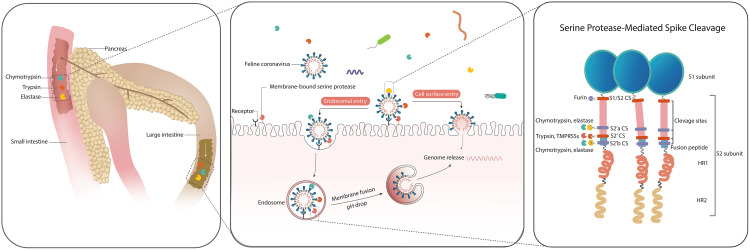
Proposed model of FCoV entry and spike protein cleavage. The three pancreatic serine proteases (trypsin, chymotrypsin, and elastase) are secreted by the pancreas and activated in the intestine. Within this intestinal environment, feline coronavirus (FCoV) employs two distinct entry pathways into host cells: the endosomal route and the cell surface route. During viral biosynthesis, the spike protein is pre-cleaved at the S1/S2 cleavage site by furin in the Golgi. A second cleavage event is required at the S2′ region to activate membrane fusion. This cleavage can be mediated either by soluble serine proteases (trypsin, chymotrypsin, elastase) or by membrane-bound proteases (TMPRSS2 and TMPRSS11D). In the endosomal pathway, cleavage occurs in the endosome after viral internalization, whereas in the cell surface pathway, it occurs at the plasma membrane. Trypsin and TMPRSS2/11D primarily target the canonical S2′ site, while chymotrypsin and elastase cleave at two distinct sites flanking S2′, referred to as S2′a CS and S2′b CS. CS: cleavage site.

Based on these observations, together with results from other coronaviruses, we propose a model for FCoV entry, as illustrated in Fig 9. Serine proteases secreted by the pancreas, trypsin, chymotrypsin, and elastase, are transported to and activated in the intestine. FCoV, which carries a S protein pre-cleaved at the S1/S2 site, hijacks either luminal pancreatic serine proteases or membrane-bound serine proteases to facilitate viral entry. Under neutral or alkaline extracellular conditions, FCoV primarily enters cells via endocytosis following receptor binding. Within the endosome, the S protein is cleaved and activated by serine proteases. As the pH decreases, membrane fusion is triggered, leading to the release of the viral genome into the cytoplasm. In contrast, under acidic extracellular conditions, spike processing by serine proteases at the plasma membrane allows direct membrane fusion at the cell surface. Trypsin and TMPRSS2/11D cleave at the S2′ CS, while chymotrypsin and elastase cleave at regions both upstream and downstream of S2′ CS named S2’a and S2’b. When S2′ is disrupted by mutation, trypsin shifts to cleave at alternative upstream and downstream sites S2’-alt1 and S2’-alt2, and TMPRSS2/11D targets the S2’-alt2 site. Considering that the pH in the intestines is typically neutral to slightly alkaline [53], FCoV is likely to enter host cells predominantly through the endosomal route. However, under pathological conditions such as colitis or local inflammation, the luminal pH can become more acidic [54]. This shift in pH allows the virus to fuse directly at the cell surface, potentially accelerating infection under pathological conditions [55].

The robust inhibitory action of camostat against FCoV infection, combined with its low cellular toxicity, is highly impressive. While various antiviral drugs targeting FCoV have been documented [56], the use of serine protease inhibitors as a strategy to combat FCoV infection has not been explored yet. This approach holds the potential to become a promising new class of antiviral treatments for cats. Baf also significantly reduces FCoV infection by blocking the endocytic entry pathway. When used in combination, camostat and Baf exhibit a striking synergistic effect *in vitro*, lowering infection rates by over 95%, suggesting a potent combinatorial approach for FCoV therapy.

## Materials and methods

### Ethics statement

Fecal samples were collected non-invasively from litter boxes in animal shelters. Approval for sample collection was obtained from the shelter management. Colonic content samples were obtained post-mortem from deceased cats, through licensed veterinarians and with the owners’ consent. The cats had died or been euthanized due to chronic diseases, and no animals were euthanized specifically for the purposes of this study. All procedures were conducted under strict biosafety protocols. As the study did not involve animal experimentation, no additional ethical approval was required.

### Cell lines, virus, proteases and inhibitors

In house produced feline intestinal epithelial cells (FIECs) [20]. were cultured in a 1:1 mixture of Dulbecco’s modified Eagle’s medium (DMEM; 61965059, Gibco) and Ham’s F-12 Nutrient Mix (11765054, Gibco), supplemented with 5% heat-inactivated fetal bovine serum (FBS), 1% penicillin-streptomycin (15140122, Gibco), 0.5% gentamicin (15710049, Gibco), and 1% non-essential amino acids (11140035, Gibco). Three serotype I FCoV strains (UCD, UG-FH8, and ABA) were propagated in FIECs with FBS-free medium, and the third-passage was used for all infection experiments. The UCD strain was originally collected at UC Davis [57] and kindly provided by Dr. Rottier (Department of Infectious Diseases and Immunology, Utrecht University). The UG-FH8 strain was isolated in 2013 at Ghent University, Belgium [20], and the ABA strain was isolated in 2019 at Ghent University, Belgium, from feces of FCoV-positive shelter cats (full name: 03.01.15_BSH_F_RU2017_2019-02-04). The human embryonic kidney 293T cell line (HEK293T, purchased from ATCC) was transfected to produce wild-type and mutated FCoV spike proteins. Cells were maintained in DMEM supplemented with 10% FBS, 1% sodium pyruvate (11360039, Gibco), 1% penicillin-streptomycin and 0.5% gentamicin.

All reagents were obtained from commercial sources. *Vibrio cholerae* neuraminidase (NA) was purchased from Roche (11080725001). Protease inhibitors, obtained from Sigma-Aldrich unless otherwise specified, were dissolved in ultrapure (UP) water or dimethyl sulfoxide (DMSO) as a stock solution as follows: 4-(2-aminoethyl)benzenesulfonyl fluoride hydrochloride (AEBSF; 208 mM in UP, A8456), camostat mesylate (50 mM in DMSO, SML0057), decanoyl-RVKR-CMK (CMK; 10 mM in DMSO, 344930), (2S,3S)-trans-Epoxysuccinyl-L-leucylamido-3-methylbutane ethyl ester (E-64d; 10 mM in DMSO, E8640), marimastat (60 mM in DMSO, M2699), and pepstatin A (1.5 mM in DMSO with 10% acetic acid, P5318). Acidification inhibitors included ammonium chloride (NH₄Cl; 1 M in UP, VWR, 12125-02-9) and bafilomycin A1 (1 mM in DMSO, MedChemExpress, 88899-55-2). Proteolytic enzymes, including L-1-tosylamide-2-phenylethyl chloromethyl ketone (TPCK)-treated trypsin (T1426), 1-chloro-3-tosylamido-7-amino-2-heptanone (TLCK)-treated chymotrypsin (MedChemExpress, HY-108910B), and pancreatic elastase (E7885), were each dissolved in 10 mM HCl containing 2 mM CaCl_2_ to a final stock concentration of 2 mg/mL. Clathrin-mediated endocytosis inhibitor chlorpromazine hydrochloride (Sigma Aldrich, C8138) was dissolved in DMSO as a stock of 140 mM.

### Cell viability assay

FIECs were seeded in 96-well plates and incubated for 24 hours. The monolayers were then treated with individual inhibitors for another 24 hours in FBS-free medium. Subsequently, 25 μL of MTT solution (5 mg/mL) was added to each well and incubated for 6 hours to allow formazan crystal formation. After removing the supernatant, 100 μL of DMSO was added to dissolve the formazan crystals, followed by gentle agitation to ensure complete dissolution. Absorbance was measured at 540 nm. Each experiment was performed in quintuplicate and repeated three times.

### Infection inhibition assay

FIEC monolayers were pretreated with 2 mU/mL NA at 37°C for 1 hour to enhance viral infection. Following NA treatment, the cells were incubated with specific protease or pH drop inhibitors for 2 hours. The concentrations of the inhibitors were as follows: AEBSF (1 μM, 10 μM), CMK (1 μM, 10 μM), Camostat (1 μM, 10 μM), E-64d (2 μM, 20 μM), Marimastat (0.05 μM, 0.5 μM), Pepstatin A (0.1 μM, 1 μM), Bafilomycin A1 (0.1 μM, 1 μM), and NH₄Cl (0.5 mM, 5 mM). All inhibitors were diluted in FBS-free medium from their respective stock concentrations. Subsequently, the cells were inoculated with three type I FCoV strains (UCD, UG-FH8, and ABA) at an multiplicity of infection (MOI) of 0.05, in the continuous presence of the inhibitors. After 1 hour of viral adsorption, the inoculum was removed, and the cells were further incubated with fresh FBS-free medium containing the inhibitors. At 12 hours post-infection (hpi), the cells were fixed, and viral infection levels were evaluated by immunofluorescence (IF) staining.

### Colonic and fecal serine protease zymography

Three colonic samples were collected from deceased cats during routine autopsies by the veterinary pathology department (Cat 1–3), and three fecal samples were collected from shelter cats (Cat 4–6). These samples were used for subsequent analysis of serine protease activity. A 10% (w/v) and 1% (w/v) suspension of colonic contents/feces was prepared in 50 mM Tris-HCl (pH 7.5). The mixture was gently shaken for 10 minutes and centrifuged at 13,000 rpm for 15 minutes. The supernatant was collected and mixed with 5x SDS-PAGE loading buffer, then loaded onto a 10% SDS-PAGE gel containing 1 mg/mL gelatin. After electrophoresis, the gel was washed twice in 50 mM Tris-HCl (pH 7.5), 2.5% Triton X-100, and 5 mM CaCl_2_ for 30 minutes to remove SDS, followed by incubation in 50 mM Tris-HCl (pH 7.5) with 5 mM CaCl_2_ for 3 hours at 37°C to allow protease activity.

The gel was stained with 40% methanol, 10% acetic acid, and 0.5% Coomassie Brilliant Blue R-250 for 45 minutes, then destained using 40% methanol and 10% acetic acid until clear bands indicating protease activity were visible. The interesting bands were excised and sent for mass spectrometry analysis to the Flemish Institute for Biotechnology (VIB) to identify the proteins. Briefly, gel was washed sequentially with water, 50% acetonitrile, and acetonitrile alone, then vacuum-dried. Proteins were digested overnight at 37 °C with MS-grade trypsin in 50 mM ammonium bicarbonate. The resulting peptides were acidified with formic acid, transferred to MS vials, and dried. Peptides were reconstituted in 0.1% TFA in 98:2 water/acetonitrile and analyzed using an Ultimate 3000 RSLC nanoLC coupled to a Q Exactive mass spectrometer. Peptide separation was performed on a C18 µPAC column using a stepped gradient, and data were acquired in data-dependent mode targeting the five most intense precursors. MS1 scans were acquired at 70,000 resolution, followed by MS/MS scans at 17,500 resolution. Instrument performance was monitored using QCloud. Data analysis was conducted via Novor Cloud with de novo sequencing and database searches. Searches used trypsin specificity, a 25 ppm precursor tolerance, and 0.02 Da fragment tolerance. Variable modifications included carbamidomethylation (C), oxidation (M), and deamidation (N). Searches were performed against three databases: UniProt, a combined Felis catus and bacterial database, and a combined Felis catus and bacterial serine protease database (taxon ID 2; downloaded March 3, 2025).

### Treatment of pancreatic serine proteases

The susceptibility of FIECs to FCoV infection was found to decline progressively with increasing passage number, likely due to downregulation of essential viral entry factors. To establish a low-susceptibility model that could highlight the enhancing effect of serine protease treatment, we selected FIECs at later passages (beyond passage 30) for this assay. FIEC monolayers, pre-treated with 2 mU/mL NA, were inoculated with the type I FCoV strains UCD, UG-FH8, and ABA at an MOI of 0.005 for 1 hour in the presence of serine proteases chymotrypsin, trypsin or elastase. After viral adsorption, the inoculum was removed, and the cells were further incubated in fresh FBS-free medium containing the respective proteases. At 12 hpi, the cells were fixed, and infection levels were assessed by IF staining.

### Immunofluorescence staining of infected enterocytes

Cells were fixed at 12 hpi (one replication cycle) with 4% paraformaldehyde for 10 minutes, followed by permeabilization with 0.1% Triton X-100 for 2 minutes. After blocking with 10% negative goat serum (NGS), cells were incubated at 37 °C for 1 hour with a mouse IgG1 monoclonal antibody 10A12, raised against the nucleocapsid (N) protein of FCoV and produced and characterized in our laboratory [58]). Subsequently, cells were incubated with a FITC-conjugated goat anti-mouse IgG secondary antibody (F-2761, Invitrogen) at 37 °C for 1 hour. Nuclei were then stained with Hoechst 33342 (H1399, Invitrogen) for 10 minutes at 37 °C. Finally, the slides were mounted using glycerol containing the anti-fading agent 1,4-diazabicyclo[2.2.2]octane (DABCO) and analyzed by a laser scanning confocal microscope (LSCM, Leica).

### Expression of membrane-bound serine proteases on FIECs

Two feline membrane-bound serine proteases, TMPRSS2 and TMPRSS11D, were expressed in FIECs. Total RNA was extracted from feline tissues using the RNeasy Mini Kit (74104, Qiagen) and reverse-transcribed into cDNA using the SuperScript First-Strand Synthesis System (11904018, Invitrogen). The coding sequences of feline TMPRSS2 and TMPRSS11D were PCR-amplified and cloned into a lentiviral expression plasmid (pLV(Exp)-EF1a) containing C-terminal V5 and His epitope tags. The sequences have been deposited in GenBank under accession numbers PV133021 (TMPRSS2) and PV133022 (TMPRSS11D).

Recombinant lentiviruses were produced by co-transfecting HEK293T cells with the lentiviral transfer plasmid constructs and packaging plasmids (psPAX2 and pMD2.G; CPCP-K2A-CT, Biocat) using Xfect Transfection Reagent (631317, Takara). Viral supernatants were collected and used to transduce FIECs in the presence of 8 μg/mL polybrene (TR-1003-G, Sigma-Aldrich). Stable polyclonal cell lines were selected using 200 μg/mL hygromycin (ant-hg-1, Invivogen) and validated for protease expression.

### Immunofluorescence staining of infected enterocytes

Cells were fixed at 12 hpi (one replication cycle) with 4% paraformaldehyde for 10 minutes, followed by permeabilization with 0.1% Triton X-100 for 2 minutes. After blocking with 10% negative goat serum (NGS), cells were incubated at 37 °C for 1 hour with a mouse IgG1 monoclonal antibody 10A12, raised against the nucleocapsid (N) protein of FCoV and produced and characterized in our laboratory [58]). Subsequently, cells were incubated with a FITC-conjugated goat anti-mouse IgG secondary antibody (F-2761, Invitrogen) at 37 °C for 1 hour. Nuclei were then stained with Hoechst 33342 (H1399, Invitrogen) for 10 minutes at 37 °C. Finally, the slides were mounted using glycerol containing the anti-fading agent 1,4-diazabicyclo[2.2.2]octane (DABCO) and analyzed by a laser scanning confocal microscope (LSCM, Leica).

### Detection of TMPRSS2 and TMPRSS11D

#### Immunofluorescence staining.

The TMPRSS2 or TMPRSS11D stably expressing FIECs were seeded in wells of a 24-well plate with inserts. After forming a monolayer, the cells were fixed with 4% paraformaldehyde for 10 min and for some of the wells followed by permeabilization using 0.1% triton X-100 for 2 min. Cells were incubated with V5 tag monoclonal antibody (R960-25, Thermofisher) containing 10% NGS at 37 °C for 1h followed by incubation with FITC-conjugated goat anti-mouse IgG secondary antibody (F-2761, Invitrogen) at 37 °C for 1h, after which cell nuclei were stained with Hoechst 33342 for 10 min at 37 °C. Slides were mounted with glycerol with DABCO and analysed by LSCM.

#### Western blot.

FIECs, stably expressing TMPRSS2 or TMPRSS11D, were seeded in a 24-well plate. After forming a monolayer, the cells were washed with ice-cold PBS and lysed with RIPA buffer (50 mM Tris, 150 mM NaCl, 5 mM EDTA, 1% Triton X-100, and a cocktail of protease inhibitors (4693159001, Merck)) under constant agitation for 30 min at 4°C. The lysates were then centrifuged at 13,000 xg for 20 min at 4°C. Supernatants were mixed with 5x Laemmli buffer and boiled for 5 min before being subjected to 10% SDS-PAGE. Proteins were transferred onto a PVDF membrane (10600023, Cytiva) and detected using a V5 tag monoclonal antibody, followed by incubation with an HRP-conjugated polyclonal goat anti-mouse antibody (P0447, Dako).

### Infection of stably TMPRSS2/11D-expressing FIEC

FIECs, stably expressing TMPRSS2 or TMPRSS11D, were seeded in a 24-well plate. After forming a monolayer, the cells were either pretreated with 2 mU/mL NA or left untreated. The cells were then inoculated with UCD, UG-FH8, or ABA at an MOI of 0.005 for 1 hour at 37 °C, after which the inoculum was removed and replaced by fresh FBS-free medium. The cells were subsequently fixed at 12, 24, and 48 hpi, and infection levels were revealed by IF staining.

### Construction of FCoV spike plasmid

The spike sequence of the type I FCoV strain ABA was selected, with its native signal peptide (SP) replaced by the tissue plasminogen activator (tPA) SP, and the transmembrane (TM) and cytoplasmic tail (CT) domains substituted with a foldon trimerization domain and a C-terminal V5/His tag. Codon optimization was implemented using the Gensmart codon optimization tool (Genscript), which was sent to GENEWIZ for synthesis. The synthesized sequence was then cloned into the pcDNA 3.1 V5/His plasmid using KpnI and ScaII restriction enzymes.

Three mutated spike plasmids were generated via site-directed mutagenesis. In plasmid MS1/S2, the S1/S2 CS was mutated (^790^RRNRRS^795^ to ^790^AANAAS^795^); in plasmid MS2’, the S2’ CS was mutated (^978^RRS^980^ to ^978^AAS^980^); in the 2M plasmid, both CSs were mutated.

### Cleavage of the S protein by serine proteases

#### Cleavage of the S protein by pancreatic serine proteases.

Subconfluent HEK293T cells (70% confluency) were transfected with four spike constructs using the Xfect transfection reagent. At 12 hours post-transfection (hpt), the culture medium was replaced by FBS-free HEK293T medium. At 48 hpt, the supernatant was collected and sequentially centrifuged at 300xg for 5 minutes, 2,000xg for 10 minutes, and 12,000xg for 15 minutes to remove cellular debris. Cells were washed three times with pre-chilled PBS, scraped using a cell scraper in FBS-free HEK293T medium, and subjected to ultrasonic disruption, followed by a centrifugation at 13,000 xg for 20 min at 4°C. Both the collected supernatant and cell lysates were treated with 2 μg/mL or 10 μg/mL serine proteases (chymotrypsin, trypsin, or elastase) at 37°C for 30 min. Samples were then mixed with non-reducing Laemmli loading buffer, boiled for 5 min, and subjected to SDS-PAGE. Cleaved fragments were detected by western blot using the V5 tag monoclonal antibody, followed by incubation with a HRP-conjugated polyclonal goat anti-mouse IgG antibody.

#### Cleavage of S protein by proteases in colonic contents.

The FBS-free supernatant from spike-transfected HEK293T cells was collected and sequentially centrifuged at 300xg for 5 minutes, 2,000xg for 10 minutes, and 12,000xg for 15 minutes to remove cellular debris. The supernatant was then incubated with 1% (w/v) colonic contents samples (prepared as described previously) at 37°C for 30 minutes. Samples were then mixed with non-reducing Laemmli loading buffer, boiled for 5 min, and subjected to SDS-PAGE. Cleaved fragments were detected by western blot using the anti-V5 tag monoclonal antibody, followed by incubation with a HRP-conjugated polyclonal goat anti-mouse IgG antibody.

#### Cleavage of S protein by membrane-bound serine protease.

Stable TMPRSS2/11D-expressing FIECs were seeded into a 24-well plate. At 70% confluency, cells were transfected with spike constructions. At 12 hpt, the culture medium was replaced with FBS-free FIEC medium. At 48 hpt, the supernatant was collected and sequentially centrifuged at 300xg for 5 minutes, 2,000xg for 10 minutes, and 12,000xg for 15 minutes to remove cell debris. Samples were then mixed with non-reducing Laemmli loading buffer, boiled for 5 min, and subjected to SDS-PAGE. Cleaved fragments were detected by western blot using the V5 tag monoclonal antibody, followed by incubation with a HRP-conjugated polyclonal goat anti-mouse IgG antibody.

### Production of FCoV spike-pseudotyped lentivirus

The wild-type ABA FCoV spike, including its native transmembrane and cytoplasmic domains, and three spike mutants (MS1/S2, MS2’, and 2M) were cloned into pcDNA3.1 with a C-terminal V5 epitope tag. To enhance pseudovirus production, the C-terminal 16 amino acids (RRQFENYEPIEKVHIH) were deleted as suggested [59]. HEK293T cells were co-transfected using Xfect with the following plasmids: (i) 1 µg of pcDNA3.1–Spike–V5 (envelope glycoprotein expression plasmid), (ii) 2 µg of psPAX2 (lentiviral packaging plasmid), and (iii) 3 µg of pLJM1-EGFP (lentiviral transfer plasmid). Culture supernatants were harvested 48 h post-transfection from 60 mm dishes, yielding approximately 10 mL of pseudovirus-containing medium. Fetal calf serum was removed by ultrafiltration using a 100-kDa molecular-weight-cutoff concentrator (Merck, UFC8100). The retentate was resuspended to 10 mL with DMEM, aliquoted, and stored at –70°C until use.

### Pseudotyped lentivirus entry assay

FIEC monolayers were pretreated with 2 mU/mL NA for 1h at 37°C. After pretreatment, cells were either left untreated or incubated with endosomal entry inhibitors (6 µM) for 2 h before infection. Spike-pseudotyped lentivirus was diluted 1:5 in DMEM and added to the FIEC monolayer in the presence or absence of trypsin (10 µg/mL) and with or without 6 µM endosomal inhibitors. Cells were incubated with the virus for 1 h at 37°C, after which the inoculum was removed. Monolayers were washed once and FBS-free medium containing the same inhibitor conditions was added. At 48 h post-infection, cells were fixed with 4% paraformaldehyde, and EGFP-positive cells were quantified by fluorescence microscopy. For each treatment, fluorescence images were acquired from 10 randomly selected fields, and the mean number of EGFP-positive cells was used for analysis.

### Statistical analysis

Statistical analysis was performed using SPSS software (version 29, IBM Corp., Armonk, NY, USA). Homogeneity of variance was assessed using Levene’s test. If the data met the assumptions of equal variances (p > 0.001), and passed the Shapiro–Wilk test for normality (p > 0.05), parametric analysis was conducted using ANOVA with Dunnett’s post hoc test. Statistical significance was indicated as follows: * (p < 0.05), ** (p < 0.01), and *** (p < 0.001). In case interactions between multiple factors were identified, cases were split by the latter factor prior to ANOVA. If the data failed the normality and/or homoscedasticity tests, appropriate transformations (e.g., logarithmic or square root) were applied to meet the assumptions. If transformation did not result in a normal distribution, non-parametric analysis was performed using the Kruskal–Wallis test followed by post hoc multiple comparisons.

## Supporting information

S1 Fig Cleavage of spike protein by 2 μg/mL serine proteases in cell lysates. HEK293T cells were transfected with spike constructs and cultured in serum-free medium for 48 h. Supernatants were collected and incubated with indicated concentrations of serine proteases at 37 °C for 30 min. Cleavage products were detected using anti-V5 western blot. Solid line: spike multimers; solid arrow: monomeric spike; dashed line: over-cleaved products.
(EPS)

S2 Fig Prediction of alternative trypsin cleavage sites in FCoV Spike. Spike protein sequences from three FCoV strains (UCD, UG-FH8, and ABA) were aligned in MEGA12 using the MUSCLE algorithm. The canonical S1/S2 and S2′ cleavage sites are highlighted with red boxes. The predicted alternative S2′ cleavage sites (S2′-alt1 and S2′-alt2) are indicated with blue and green boxes, respectively. Basic amino acids located between the S1/S2 and S2′ regions are shown in bold red font.
(EPS)

S3 Fig Endogenous expression of TMPRSS2/TMPRSS11D in feline tissues. Total RNA was extracted from six feline colonic tissues and feline intestinal epithelial cells (FIECs). After reverse transcription, the resulting cDNA was used as a template for PCR amplification. Expected bands are indicated by black arrows. Lanes 1–6: feline colonic tissue samples; Lane 7: FIECs.
(EPS)

S4 Fig Detection of TMPRSS2/TMPRSS11D expression in stable cell lines. (A) Immunofluorescence detection of exogenous TMPRSS2/TMPRSS11D. FIECs were transduced with lentiviral vectors encoding V5-tagged TMPRSS2 or TMPRSS11D, followed by hygromycin selection to establish stable cell lines. Cells were fixed with 4% paraformaldehyde and either permeabilized with 0.1% Triton X-100 or left non-permeabilized. TMPRSS2/TMPRSS11D proteins were detected using an anti-V5 antibody. (B) Western blot analysis of exogenous TMPRSS2/TMPRSS11D expression. Hygromycin-selected, TMPRSS2/TMPRSS11D transduced FIECs were lysed and analyzed by SDS-PAGE and western blot with an anti-V5 antibody to confirm protein expression.
(EPS)

S1 File Feline coronavirus S2′ cleavage site sequences retrieved from GenBank. This file contains amino acid sequences spanning the S2′ cleavage site from 167 Feline Coronavirus (FCoV) strains obtained from the GenBank database. The sequences were aligned and only the regions corresponding to the S2′ cleavage motif (positions P5–P1 and P1′–P5′) were retained for further analysis. These curated sequences were used for the conservation analysis of the S2′ cleavage site shown in Fig 6. All sequences are provided in FASTA format, and the corresponding GenBank accession numbers are included for reference.
(FAS)
