# Correction: Serine protease-driven entry and S2 ′ cleavage flexibility of feline coronavirus during feline enterocyte infections

**DOI:** 10.1371/journal.ppat.1014259

**Published:** 2026-05-28

**Authors:** Bixia Chen, Luna Vanden Buijs, Nathalie Vanderheijden, Lowiese Desmarets, Jolien Van Cleemput, Hans J. Nauwynck

The following panels in [1] are incorrect:

- Fig 5A:
  - WT 24 hpi
  - MS1/S2 24 hpi
  - MS2’ 5 and 24 hpi
  - 2M 5 and 24 hpi
- Fig 5B: MS2’ (- trypsin, - chlorpromazine)
- Fig 8A: -NA ABA MOCK 48 h.p.i.

Corrected Figs 5 and 8 are provided with this notice. The figure legends for these figures have also been updated to improve clarity and to define abbreviations.

The corrected figures have been reviewed by a member of the *PLOS Pathogens* Editorial Board, who stated that the conclusions in [1] remain supported.

The authors apologize for the errors in the published article.

**Fig 5:**
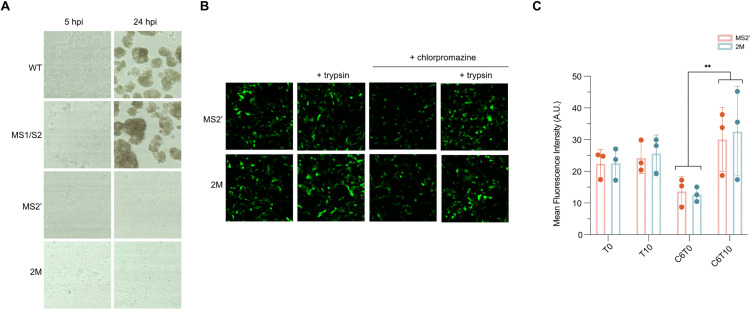
Entry of pseudotyped viruses carrying wild-type or mutant FCoV Spike. **(A)** Optical microscopy images of feline intestinal epithelial cell (FIEC) monolayers following inoculation with four Spike-pseudotyped lentiviruses (wild-type, MS1/S2, MS2’, and 2M). Neuraminidase (NA)-pretreated FIEC monolayers were inoculated with each pseudotyped virus, and images were captured at 5 and 24 hours post-infection (hpi). Cytopathic effects (CPE) were observed in cells infected with wild-type and MS1/S2 pseudoviruses, but not with MS2’ or 2M. **(B)** Representative fluorescence microscopy images of FIEC monolayers at 48 hpi following inoculation with MS2’ or 2M Spike-pseudotyped lentiviruses. Cells were pretreated with NA and infected under the following conditions: untreated, supplemented with trypsin (10 µg/mL), treated with chlorpromazine (CPZ, 6 µM), or treated with both CPZ and trypsin. **(C)** Quantitative analysis of MS2’ and 2M pseudovirus entry under the treatment conditions described in panel (B). Data represent the mean EGFP fluorescence intensity measured from 10 random fields per condition. Statistical significance was determined by two-way ANOVA followed by Tukey HSD post hoc test. F(3, 16) = 6.796, p = 0.004. ** indicates p < 0.01. T0: without trypsin; T10: 10 µg/mL trypsin; C6: 6 µM CPZ.

**Fig 8:**
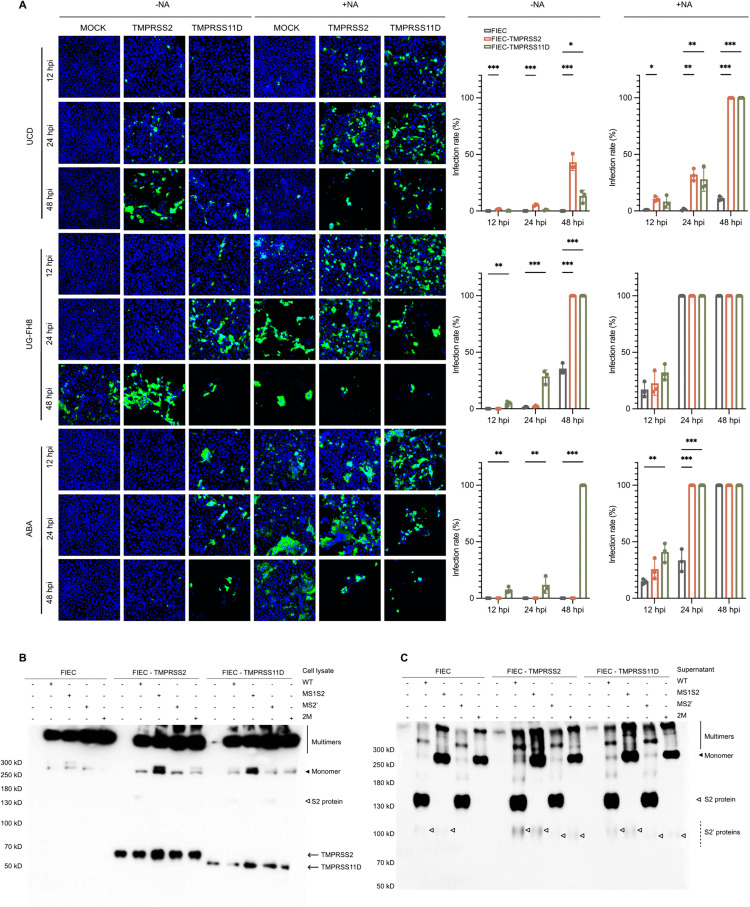
Impact of TMPRSS2 and TMPRSS11D on FCoV replication and spike cleavage. **(A)** TMPRSS2 and TMPRSS11D enhance viral infection. Feline intestinal epithelial cells (FIECs) stably expressing TMPRSS2 or TMPRSS11D were pre-treated with or without neuraminidase (NA), followed by infection with type I FCoV strains (UCD, UG-FH8, or ABA) at an MOI of 0.005. At 12, 24, and 48 hours post-infection (hpi), cells were fixed and stained with an anti-nucleocapsid antibody. Representative immunofluorescence images and quantification show increased infection rates in TMPRSS2- and TMPRSS11D-expressing cells compared to controls. Infection was quantified as the percentage of nucleocapsid-positive cells among total cells. Data represent mean ± SEM (n = 3). Statistical analysis was performed separately for each virus strain, neuraminidase treatment, and time point due to interaction effects, using one-way ANOVA F(2, 108) = 576.919 with Dunnett’s post hoc test to assess differences between cell lines. *p < 0.05, **p < 0.01, ***p < 0.001. **(B, C)** Spike cleavage in FIECs expressing TMPRSS2 or TMPRSS11D. Cells were transfected with V5-tagged spike constructs, and at 48 hours post-transfection (hpt), cell lysates (B) and supernatants (C) were collected. Cleavage products were analyzed by western blot using an anti-V5 antibody. Expression of full-length and cleaved spike fragments was evaluated to determine proteolytic activity. Solid line: spike multimers; solid arrow: monomeric spike; open arrow: S2 protein (from S1/S2 cleavage); dashed lines: S2′ proteins (from S2′ or alternative cleavages).
